# Asian Elephant T Cell Responses to Elephant Endotheliotropic Herpesvirus

**DOI:** 10.1128/JVI.01951-17

**Published:** 2018-02-26

**Authors:** Angela Fuery, Ann M. Leen, Rongsheng Peng, Matthew C. Wong, Hao Liu, Paul D. Ling

**Affiliations:** a Molecular Virology and Microbiology, Baylor College of Medicine, Houston, Texas, USA; b Center for Cell and Gene Therapy, Baylor College of Medicine, Texas Children's Hospital, Houston Methodist Hospital, Houston, Texas, USA; c Department of Biostatistics, Indiana University School of Medicine, Indianapolis, Indiana, USA; University of California, Irvine

**Keywords:** IFN-γ, ELISpot, EEHV, Asian elephant, T cell

## Abstract

Elephant endotheliotropic herpesvirus (EEHV) can cause lethal hemorrhagic disease in juvenile Asian elephants, an endangered species. One hypothesis to explain this vulnerability of some juvenile elephants is that they fail to mount an effective T cell response to the virus. To our knowledge, there have been no studies of Asian elephant T cell responses to EEHV. To address this deficiency, we validated the gamma interferon (IFN-γ) enzyme-linked immunospot assay for tracking antigen-directed T cell activity by monitoring rabies-specific responses in vaccinated elephants. In addition, we generated monoclonal antibodies to Asian elephant CD4 and CD8 to facilitate phenotypic T cell profiling. Using these tools, we screened healthy elephants with a history of EEHV infection for reactivity against nine EEHV proteins whose counterparts in other herpesviruses are known to induce T cell responses in their natural hosts. We identified glycoprotein B (gB) and the putative regulatory protein E40 as the most immunogenic T cell targets (IFN-γ responses in five of seven elephants), followed by the major capsid protein (IFN-γ responses in three of seven elephants). We also observed that IFN-γ responses were largely from CD4^+^ T cells. We detected no activity against the predicted major immediate early (E44) and large tegument (E34) proteins, both immunodominant T cell targets in humans latently infected with cytomegalovirus. These studies identified EEHV-specific T cells in Asian elephants for the first time, lending insight into the T cell priming that might be required to protect against EEHV disease, and will guide the design of effective vaccine strategies.

**IMPORTANCE** Endangered Asian elephants are facing many threats, including lethal hemorrhagic disease from elephant endotheliotropic herpesvirus (EEHV). EEHV usually establishes chronic, benign infections in mature Asian elephants but can be lethal to juvenile elephants in captivity and the wild. It is the leading cause of death in captive Asian elephants in North America and Europe. Despite the availability of sensitive tests and protocols for treating EEHV-associated illness, these measures are not always effective. The best line of defense would be a preventative vaccine. We interrogated normal healthy elephants previously infected with EEHV for T cell responses to nine EEHV proteins predicted to induce cellular immune responses. Three proteins elicited IFN-γ responses, suggesting their potential usefulness as vaccine candidates. Our work is the first to describe T cell responses to a member of the proposed fourth subfamily of mammalian herpesviruses, the Deltaherpesvirinae, within a host species in the clade Afrotheria. An EEHV vaccine would greatly contribute to the health care of Asian and African elephants that are also susceptible to this disease.

## INTRODUCTION

Elephant endotheliotropic herpesvirus (EEHV) can cause acute hemorrhagic disease in juvenile Asian elephants, with a high mortality rate. There are four species of EEHV that are endemic in Asian elephants (EEHV1A, EEHV1B, EEHV4, and EEHV5), where the majority of adults are most likely latently infected with several or all of these species (1). The two chimeric variants of EEHV1, EEHV1A and EEHV1B, cause the majority of lethal disease, in both captive and wild elephants (2). The greatest incidence of death from EEHV occurs in calves from 1 to 8 years of age, and recent estimates indicate that it is the single largest cause of death in captive juvenile Asian elephants in North America and Europe (2). Hemorrhagic disease caused by EEHV is associated with a large viral burden, suggesting that uncontrolled infection plays a role in causing the disease. Why some elephants succumb to lethal infection remains unknown, but we hypothesize that one factor could be a failure to mount an effective cellular immune response. Insufficiencies in cell-mediated immunity are a major risk factor in other herpesvirus-associated infections and reactivation in humans (cytomegalovirus [CMV] [3–7], varicella-zoster virus [VZV] [8, 9], Epstein-Barr virus [EBV] [10], and human herpesvirus type 6 [HHV-6] [11]). Hence, there is a distinct need to understand the T cell response to EEHV in Asian elephants in order to find a solution to the devastating effects of this lethal virus.

To date, Asian elephant T cell responses at the cellular level have not been explored, in part because there are limited reagents to detect phenotypic (cell surface markers) and functional characteristics (Asian elephant cytokines). In the present study, we developed Asian elephant-specific reagents and methods, which we have used to elucidate the cellular immune response to EEHV. We have studied a herd of latently infected elephants with a normal pattern of reactivation and control (12, 13) in order to identify the specificity and functional profile of protective T cells. The sequenced genome of EEHV1A has identified approximately 115 open reading frames (ORFs), 37 of which are conserved core genes, common to all herpesviruses, and 15 of which are conserved within the betaherpesvirus and gammaherpesvirus subfamilies (14, 15). Among these conserved proteins, several are similar to structural and regulatory proteins that have been identified as potent inducers of T cell responses in humans. Thus, we synthesized overlapping peptide libraries (15mers overlapping by 11 amino acids) spanning nine EEHV ORFs, which we used to interrogate the immune response in seven latently infected adult and juvenile elephants. Now, using a panel of unique elephant-specific reagents, we report on the first T cell immune responses directed against EEHV, which should assist in the design of future vaccines or T cell therapies and the evaluation of their efficacy.

## RESULTS

### Rabies-specific gamma interferon (IFN-γ) responses are detectable within the peripheral blood of Asian elephants following routine vaccination.

**FIG 1:**
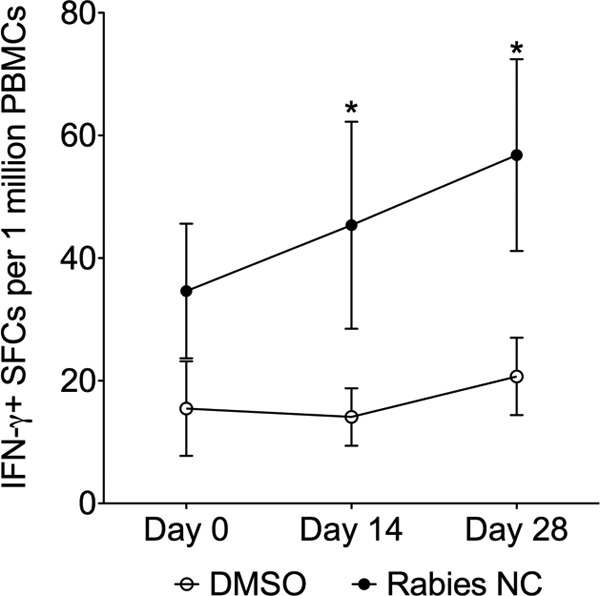
IFN-γ ELISpot following rabies vaccine. Five elephants were vaccinated with killed rabies vaccine at day 0, and blood was obtained from five elephants (aged 9 to 49) at days 14 and 28. PBMCs were stimulated in IFN-γ-coated ELISpot plates with DMSO control or rabies NC pepmix. Each sample was tested in triplicate at each time point in at least three separate experiments. The means ± the standard errors of the mean (SEM) of SFCs per 1 million PBMCs is shown, where “*” (*P* < 0.05) indicates a statistically significant difference as determined by two-sample *t* tests on log-transformed values compared to the DMSO control at the same time of postvaccination.

Conditions for investigating T cell responses in any animal within the clade Afrotheria have not been established. Thus, to first validate the IFN-γ enzyme-linked immunospot (ELISpot) assay as a means to assess protective T cell immune responses in elephants, we first focused on rabies as a model pathogen and specifically on measuring immunity to the rabies nucleocapsid protein (NC), which has been shown to induce cellular immune responses (16). Five of our elephants had a history of receiving a rabies vaccine as part of their regular health management and received a booster vaccination during the course of our study. To monitor T cell immunity in these animals, we utilized a pepmix (a library of consecutive 15mer peptides, overlapping by 11 amino acids) spanning rabies nucleocapsid (rabies NC) as an immunogen, as well as Asian elephant IFN-γ-specific antibody pairs originally developed for an enzyme-linked immunosorbent assay (17) and adapted here for use in the ELISpot assay. Blood was collected from each of the elephants prior to (day 0) and at days 14 and 28 after vaccination, and the frequency of reactive cells assessed after stimulation with the NC pepmix. As shown in Fig. 1, there was a significant increase in the frequency of IFN-γ-secreting cells (spot forming cells [SFCs]) at both day 14 (*, *P* = 0.018) and day 28 (*, *P* = 0.035) postvaccination compared to the control (dimethyl sulfoxide [DMSO] solvent) at the corresponding time points. In addition, we found that unlike phytohemagglutinin or phorbol myristate acetate/ionomycin, staphylococcus enterotoxin B (SEB) was able to nonspecifically activate elephant cells to secrete IFN-γ, so we incorporated SEB into our subsequent assays for use as a positive control (data not shown).

### Identification of EEHV proteins that elicit IFN-γ responses.

**FIG 2:**
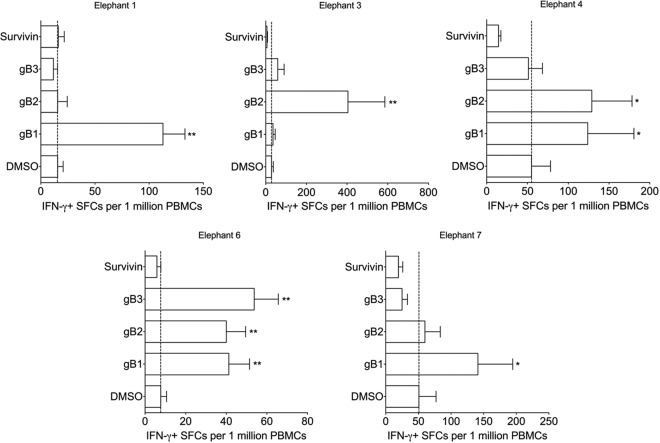
Glycoprotein B (gB) responses in five elephants. PBMCs obtained from each elephant at three separate time points were screened in triplicate in at least three separate experiments. The means the ± SEM of SFCs per 1 million PBMCs is shown, where “*” (*P* < 0.05) and “**” (*P* < 0.01) indicate statistically significant differences determined by two-sample *t* tests on log-transformed values compared to the DMSO control. Survivin is included as an additional negative control. The dashed line represents the mean of DMSO (background) for each elephant.

**FIG 3:**
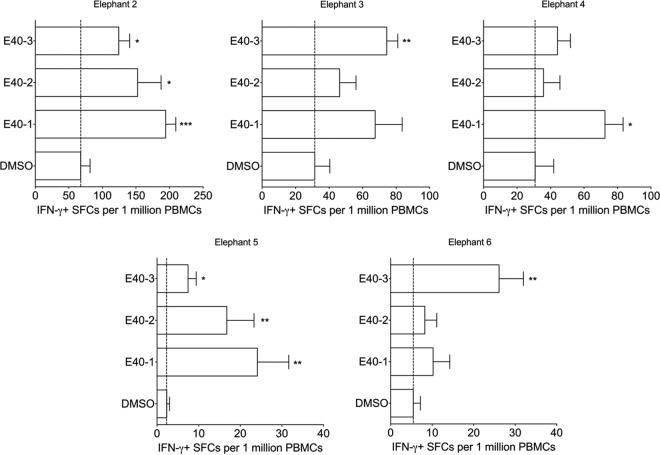
E40 responses in five elephants. PBMCs obtained from each elephant at three separate time points were screened in triplicate in at least three separate experiments. The means ± the SEM of SFCs per 1 million PBMCs is shown, where “*” (*P* < 0.05), “**” (*P* < 0.01), and “***” (*P* < 0.001) indicate statistically significant differences as determined by two-sample *t* tests on log-transformed values compared to the DMSO control. The dashed line represents the mean of DMSO (background) for each elephant.

**FIG 4:**
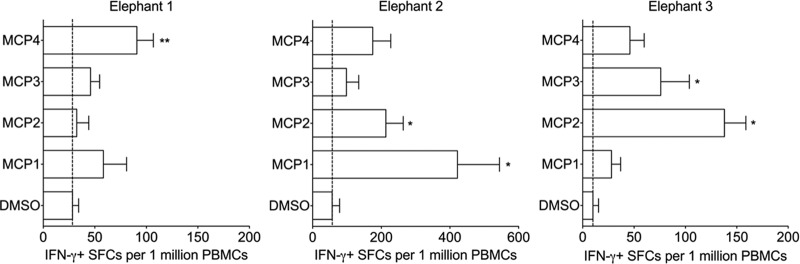
Major capsid protein (MCP) responses in three elephants. PBMCs obtained from each elephant at three separate time points were screened in triplicate in at least 3 separate experiments. The means ± the SEM of SFCs per 1 million PBMCs is shown, where “*” (*P* < 0.05) and “**” (*P* < 0.01) indicate statistically significant differences as determined by two-sample *t* tests on log-transformed values compared to the DMSO control. The dashed line represents the mean of DMSO (background) for each elephant.

Having established the IFN-γ ELISpot as an effective means to detect Asian elephant antigen-specific T cell responses, we applied this approach to detect immune responses to selected proteins of EEHV1A, which has been associated with the largest number of deaths caused by EEHV. Thus, we characterized responses to nine predicted EEHV1A proteins, which are described in Table 1. These proteins were selected largely because they share characteristics with other herpesvirus proteins that have been shown to elicit robust T cell responses (Table 1). Based on the sequence information from EEHV1A strain Kimba, we synthesized individual 15mer peptides, overlapping by 11 amino acids and arranged them into ORF-specific mixes or, for larger ORFs, into sub-ORF mixes of approximately 60 to 90 peptides and subsequently used these pepmixes to screen peripheral blood mononuclear cells (PBMCs) isolated from seven elephants (Table 2) by IFN-γ ELISpot assay. Although nine ORFs were studied, we detected significant responses to three: gB (five elephants; Fig. 2), E40 (five elephants; Fig. 3), and MCP (three elephants; Fig. 4). Each of these figures shows responses to sub-ORF mixes compared to the negative control DMSO. Survivin was used as an additional negative control in early studies (Fig. 2); however, responses to survivin were generally lower than DMSO, so we chose DMSO as a more conservative control for most of our studies (Fig. 3 and 4). Table 3 summarizes all nine ORFs and the responses induced in each elephant screened.

**TABLE 1:** Summary of EEHV1A ORFs selected for screening in IFN-γ ELISpot assays

ORF(s)^*a*^	Common name(s)	Protein type	Homolog(s)	T cell reference(s)
U57	Major capsid protein (MCP)	Capsid/structural	HHV6 U57, HCMV UL86, HSV UL19	25, 26
E44, EE1*	ORF-L, major immediate early	Regulatory	HCMV UL123	
U39	gB	Glycoprotein	HCMV UL55	23, 24
U48	gH	Glycoprotein	HHV6 U48, HCMV UL75, HSV UL22	26
U42	ICP27, Mta	Regulatory	HHV6 U42, HCMV UL69, HSV UL54	41
E34, U11*	ORF-C, pp150	Tegument/structural	HCMV UL32	
U71	TP Myrs Teg	Tegument/structural	HHV6 U71, HCMV UL99, HSV UL11	42
E40, EE2*	ORF-K	Nuclear protein	Unknown	
E44A, EE1A*	ORF-S	Putative regulatory	Different reading frame from ORF-L	

a *, Alternative nomenclature used in Wilkie et al. (15).

**TABLE 2:** Characteristics of the Houston Zoo elephant herd

Elephant	Age (yrs)	Sex	EEHV1 characteristic (source or reference)	
			Viremia^*a*^	Shedding (trunk wash)
1	52	M	Yes (unpublished data)	Yes (unpublished data)
2	48	F	Yes (unpublished data)	Yes (13)
3	27	F	Yes (12)	Yes (12, 13)
4	36	F	Yes (unpublished data)	Yes (13)
5	12	M	Yes (unpublished data)	Yes (unpublished data)
6	7	M	Yes (44)	Yes (44)
7	7	F	Yes (44)	Yes (44)
8	3	M	No	No
9	0	F	No	No

a Indicating the detection of EEHV1 at least once in whole blood samples screened weekly for EEHV from 2009 to 2017.

**TABLE 3:** Responses to EEHV1A ORFs studied

ORF^*a*^	Response^*b*^ in elephant:						
	1	2	3	4	5	6	7
U57	**+**	**+**	**+**	–	–	–	–
E44, EE1*	–	–	–	–	–	–	–
U39	**+**	–	**+**	**+**	–	**+**	**+**
U48	–	–	–	–	–	–	–
U42	–	–	–	–	–	–	–
E34, U11*	–	–	–	–	–	–	–
U71	–	–	–	–	–	–	–
E40, EE2*	–	**+**	**+**	**+**	**+**	**+**	–
E44A, EE1A*	–	–	–	–	–	–	–

a *, Alternative nomenclature used in Wilkie et al. (15).

b Responses are scored as detected (+) or not detected (–).

**FIG 5:**
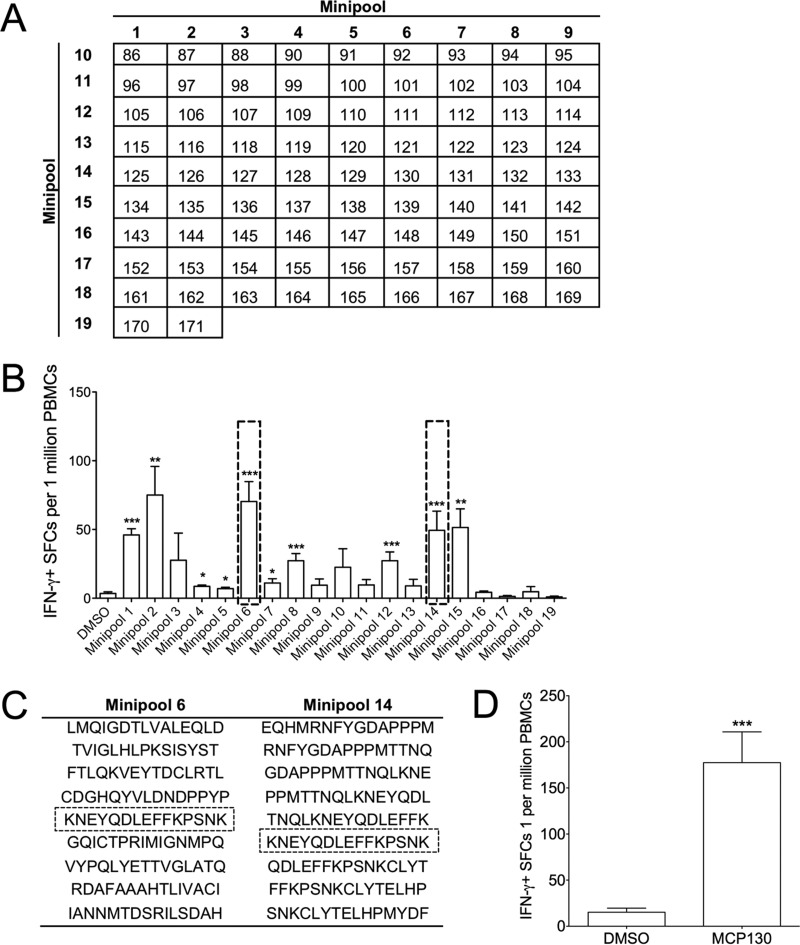
Deconvolution of MCP2 peptide pool. Elephant 3, who had a significant IFN-γ ELISpot response to MCP2, was selected for further study with the intention of identifying the peptide sequence within MCP2 to which the T cells were responding. (A) MCP2 matrix summary. Numbers on the top row and in the first column indicate minipool numbers, and each number in the grid represents an individual peptide. (B) MCP2 minipool summary showing the means ± the SEM of SFCs per 1 million PBMCs, where “***” (*P* < 0.001), “**” (*P* < 0.01), and “*” (*P* < 0.05) indicate statistically significant differences as determined by two-sample *t* tests on log-transformed values compared to the DMSO control. Boxes were placed around minipools 6 and 14, which intersected at MCP130. (C) Sequences of nine peptides in minipools 6 and 14 with boxes around the sequence of MCP130 demonstrate its presence in both minipools. (D) PBMCs from elephant 3 were further stimulated with MCP130 alone, where there was a significant response above the DMSO control (***, *P* < 0.001).

Sufficient PBMCs from one elephant (elephant 3) who responded significantly to MCP were available to deconvolve some of the larger peptide mixes. Thus, we generated minipools as described previously (18) (Fig. 5A), which we used individually to stimulate elephant PBMCs. As shown in Fig. 5B, we detected activity against multiple stimulating minipools, suggesting activity against numerous MCP peptides. This was confirmed for MCP130 (AA 517-531, sequence KNEYQDLEFFKPSNK, present in minipools 6 and 14; Fig. 5B and C), which induced strong IFN-γ-specific activity when used to stimulate PBMCs from elephant 3 (Fig. 5D).

### Phenotype of EEHV-peptide-specific T cell responses.

**FIG 6:**
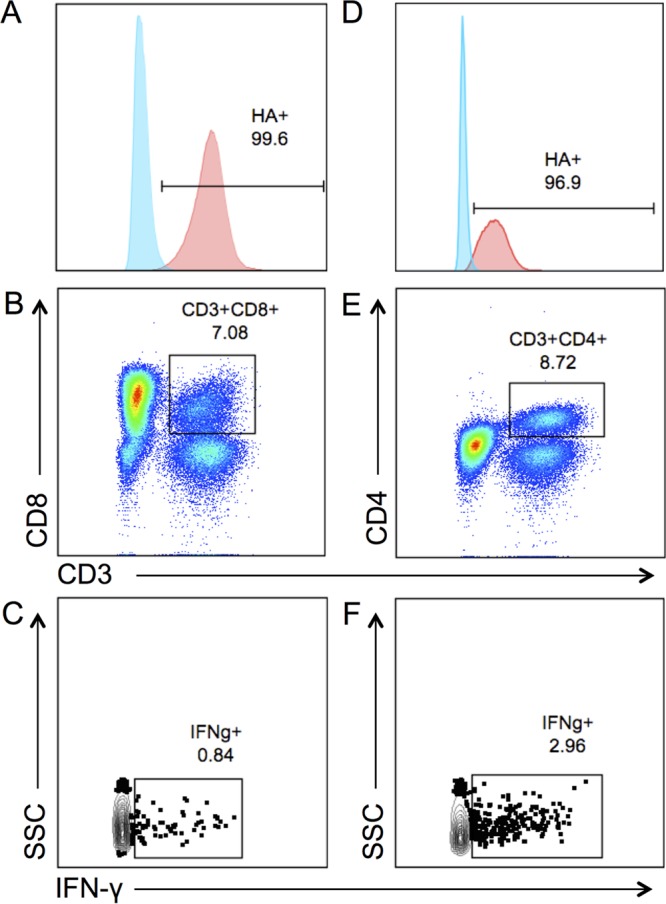
CD8 and CD4 protein expression in L cells and PBMCs. CD8 and CD4 expression was identified in L cells with primary antibodies directed against the HA epitope tag. No CD8 or CD4 expression was seen in CD8^−^ and CD4^−^ L cell lines (A and D, respectively) compared to broad expression in CD8^+^ and CD4^+^ cell lines (A and D). Using purified monoclonal antibodies on elephant PBMCs in combination with a cross-reactive CD3 monoclonal antibody, we were able to detect CD3^+^ CD8^+^ T cells (B) and CD3^+^ CD4^+^ T cells (E). Both CD3^+^ CD8^+^ and CD3^+^ CD4^+^ subsets can be further quantified as IFN-γ producers using an IFN-γ monoclonal antibody, shown here in cells stimulated with SEB (C and F).

To determine the phenotype of EEHV-reactive T cells, we first identified a human CD3 monoclonal antibody that recognizes Asian elephant CD3 at a highly conserved site on the epsilon chain of CD3. To further understand whether responding T cells were helper (CD4^+^) or cytotoxic (CD8^+^), we generated Asian elephant CD8- and CD4-directed monoclonal antibodies, by first creating mouse L cell lines expressing predicted African elephant CD8 and Asian elephant CD4 proteins (Fig. 6A and D). Each L cell line was then used to inoculate mice to generate hybridomas, which were screened and characterized as described previously (19). Supernatants were tested on Asian elephant PBMCs to confirm their ability to detect actual protein expression, and successful hybridoma clones were then subcloned prior to making purified antibody which, in combination with a CD3 monoclonal, enabled the discrimination of CD8^+^ and CD4^+^ T cells (Fig. 6B and E). Furthermore, CD8^+^ and CD4^+^ IFN-γ-secreting populations could be detected by intracellular cytokine staining after the activation of Asian elephant PBMCs with SEB (Fig. 6C and F).

**FIG 7:**
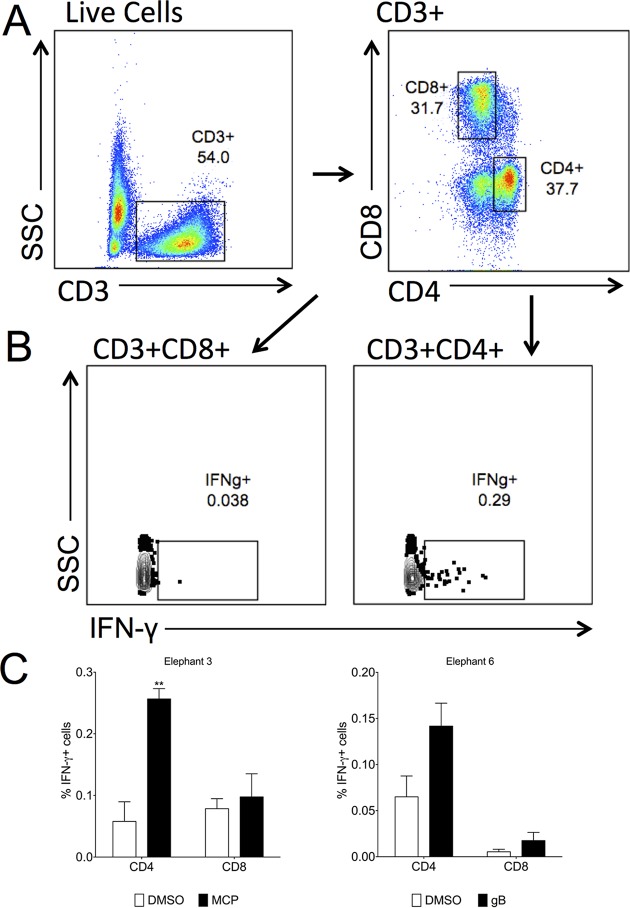
Determining T cell phenotype after peptide stimulation. PBMCs were surface stained with Asian elephant CD8- and CD4-specific monoclonal antibodies and stained for intracellular CD3 and IFN-γ expression after peptide stimulation. (A) After the exclusion of dead cells and doublets, the cells were gated on CD3 expression prior to CD4 and CD8 expression. (B) The IFN-γ expression of each CD3^+^ CD8^+^ and CD3^+^ CD4^+^ fraction is shown for one elephant who had a specific response to MCP130. (C) The frequency of IFN-γ expression in CD3^+^ CD8^+^ and CD3^+^ CD4^+^ cells from two elephants was assessed by fluorescence-activated cell sorting. **, *P* < 0.01.

Finally, we combined the cross-reactive CD3 monoclonal antibody and our custom-made CD8 and CD4 monoclonal antibodies to determine the nature of the IFN-γ response after stimulation with EEHV peptides via flow cytometry. We initiated our studies using the peptide MCP130, which induced strong responses in elephant 3 (Fig. 5). As shown in Fig. 7, we were able to detect both CD4^+^ (37.7%) and CD8^+^ populations (31.7%) in PBMCs (Fig. 7A). However, after peptide stimulation, only CD3^+^ CD4^+^ cells produced IFN-γ (0.29%), whereas CD3^+^ CD8^+^ cells were unresponsive (0.038%), indicating that MCP130 contains a CD4^+^ T cell epitope (Fig. 7B). Similarly, in PBMCs from two elephants who had robust responses to gB and MCP, as detected by IFN-γ ELISpot assay, we detected dominant responses in the CD4^+^ T cell compartment after antigen stimulation (Fig. 7C).

## DISCUSSION

This study has for the first time demonstrated the detection of EEHV-specific T cells in the peripheral blood of latently infected Asian elephants using an IFN-γ ELISpot assay expressly established and validated (using rabies as a model pathogen) to facilitate immunologic profiling. In seven elephants screened for activity against nine antigens predicted to be immunogenic based on studies of homologs from other herpesviruses, we identified glycoprotein B, E40, and the major capsid protein as being immunodominant, eliciting immune responses predominantly in the CD4^+^ T cell compartment. These data provide the first clues to the types of T cell responses that might be required to prevent EEHV hemorrhagic disease in juvenile elephants and preliminarily identify potential EEHV vaccine candidate proteins.

After primary infection EEHV establishes a lifelong latency, with occasional reactivation as demonstrated by intermittent shedding from mucosal surfaces such as the trunk and in saliva (12, 13). Based on routine monitoring of trunk wash samples or prior detection of viremia, we confirmed that all but two juveniles in the Houston zoo herd were latently infected with EEHV1 (Table 2). Hence, we were assured that a T cell response specific to EEHV1A proteins would be measurable in this herd if it existed and if appropriate tools were available. Not knowing the dynamics of EEHV-specific T cell immunity, we first established our assays using rabies, a killed whole virus vaccine known to induce protective T cell immunity (20, 21). Indeed, consistent with published studies, we were able to detect IFN-γ-producing T cells after stimulation with rabies NC, one of the five encoded proteins (22), which significantly increased in number postvaccination. These results confirm the effectiveness of the IFN-γ monoclonal antibodies and the sensitivity of the IFN-γ ELISpot assay for detecting circulating memory T cell responses to EEHV proteins in the peripheral blood of Asian elephants.

By utilizing our optimized ELISpot assay to assess the immunogenicity of EEHV candidate proteins, we identified three antigens—gB, MCP, and E40—that elicited significant IFN-γ responses in adult and juvenile elephants who were latently infected with EEHV. Both gB and MCP, which have homologs in HHV-6 and CMV, have been shown to elicit significant IFN-γ responses in humans (23–26). Since both are likely abundant structural proteins that are delivered upon infection to the host cell, it is not surprising that they both elicit significant T cell responses in this latently infected herd. E40, a putative regulatory protein, which elicited broad yet modest T cell responses in this herd, is a protein unique to EEHV, so we are unable to speculate as to the significance of these findings based on the expression of this protein in EEHV. However, it is not surprising that a putative regulatory protein would induce T cell responses, since regulatory proteins of other herpesviruses are well documented in eliciting T cell responses (24, 27, 28). Hence, we remain interested in further understanding responses to this novel protein.

We were able to determine the nature of the T cell response to both gB and MCP in positive elephants, where we found that responses were predominantly from CD4^+^ T cells. In previous studies of other herpesviruses, gB has elicited responses in both CD8^+^ (29, 30) and CD4^+^ (31–33) T cells, although dominant gB epitopes seem to be predominantly major histocompatibility complex (MHC) class II associated. Our results for MCP are consistent with its homolog U57 in HHV-6, responses to which are also dominated by CD4^+^ T cells (25-27, 34). The dominance of CD4^+^ responses to both gB and MCP potentially indicates their importance in forming quality antibody responses, but CD4^+^ T cells have also been implicated in assisting CD8^+^ T cell migration to sites of infection (35), and there is increasing evidence of their cytotoxic potential (31, 36–38). Having characterized a dominance of CD4^+^ T cell responses in these two positive proteins, we do not discount the potential for MHC class I epitopes in other EEHV proteins. Indeed, responses to CMV, another betaherpesvirus, have been dominated by the CD8^+^ T cell subset (39), and there is the potential for any of the remaining 106 proteins of EEHV1A not yet studied to contain MHC-I epitopes.

We saw no significant response to the six other proteins screened in this study. These proteins were selected based on the ability of similar proteins to induce robust T cell responses in studies of other herpesviruses. Most notably, we expected to see strong responses to the structural protein E34, which most closely resembles pp150 in CMV and the regulatory protein major immediate early protein E44, whose homolog UL123 (IE1) of CMV is among the most immunogenic of ORFs in CMV (24, 28). Although EEHV is broadly recognized as a betaherpesvirus like CMV and HHV-6, it has been proposed that the genetic differences between EEHV1A, EEHV1B, and EEHV2 and the other betaherpesviruses make them candidates for a potential new subfamily of deltaherpesviruses, as an intermediate branch between betaherpesviruses and gammaherpesviruses (40). The difference in responses to proposed EEHV homologs might therefore be attributable to the divergence of EEHV1A from the broad classification of a betaherpesvirus and the ultimate uniqueness of this virus.

Since this is the first study of T cell responses in Asian elephants using IFN-γ ELISpot as a primary screening tool, we were initially careful to draw parallels between our data and similar studies in other species to confirm the sensitivity of our system in Asian elephants. We are confident in our positive responses since they are within the range of similar studies in humans and in nonhuman primates (41–43). One drawback in our analysis is that we did not have access to appropriate uninfected negative-control elephants. While there are two juvenile elephants (ages 5 months and 3.5 years) in the Houston Zoo herd which are potentially naive to EEHV, obtaining sufficient blood from them to conduct ELISpot experiments remains impractical at the current time. In addition to developing the ELISpot assay for detection of antigen-specific T cells directly from peripheral blood, we also attempted to increase the sensitivity of our assay by selectively expanding EEHV-specific T cells *in vitro* using both recombinant human cytokines and elephant cytokines made in-house to support cell growth. To date, our efforts to identify a specific protocol to selectively enrich and expand reactive populations has been unsuccessful, but this is likely to change as additional elephant-specific reagents become available.

The response to gB in two juvenile elephants seems to indicate some level of antigen experience in these elephants, which may suggest that they have both already had primary EEHV1A infection. Based on routine monitoring of the herd, we are aware that one of these juveniles (elephant 7) has had a primary EEHV1A infection, but to our knowledge the susceptibility of the other one (elephant 6) to EEHV1A infection was still unknown, despite this elephant already having an EEHV1B viremia (44). The gB sequence is 79% conserved between EEHV1A and EEHV1B, so it is possible that this activity is due to cross-reactive T cell recognition, especially since this elephant also has a significant response to E40. The strength of responses to gB in these two juveniles, who are likely to have been infected more recently, rather than to MCP, which did not induce any responses in juveniles and E40, which induced a response in only one juvenile, may indicate gB is presented with higher frequency to naive T cells, due to its surface exposure. As such, gB may serve as a better vaccine candidate. Indeed, herpesvirus vaccine development has largely focused on gB as a candidate (45–48) owing to its surface exposure and the presence of gB-specific antibodies in CMV-seropositive patients (49).

A significant limitation of this study is the small sample size and hence lack of MHC diversity. In studying only seven elephants, some of which are related, it is likely that we are only detecting dominant peptide sequences that bind to a very small set of MHC molecules. Indeed, studies of human herpesviruses are far more diverse in their population sampling and many have characterized the precise MHC haplotypes associated with particular epitopes (25, 26, 28). At this stage, we can only hope to diversify our study population by screening more elephant herds until appropriate technologies to determine the MHC haplotype of the elephants are developed. For now, gauging dominant T cell epitopes irrespective of MHC haplotype in our current herd is a major step forward in understanding the nature of T cell responses to EEHV, for which no prior study of this nature has existed. In addition, we have generated two monoclonal antibodies and pioneered two elephant specific T cell assays that will be essential in downstream studies of T cell responses in Asian elephants. These steps are significant in advancing the understanding of EEHV and what might be required for future EEHV vaccine development.

## MATERIALS AND METHODS

### Study population.

**FIG 8:**
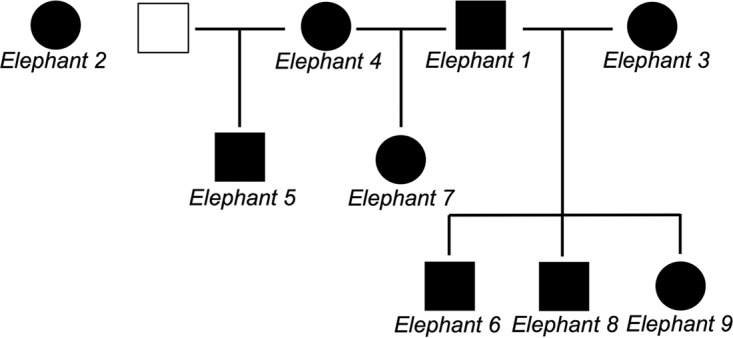
Houston Zoo elephant herd. Squares represent males, and circles represent females. One male (indicated by a white square) is not a member of the Houston Zoo herd but is the father of elephant 5. The features of each elephant are described in Table 2.

The elephants in this study belong to a single herd of nine elephants consisting of five females (ages 5 months and 7, 27, 36, and 48 years old) and four males (3, 7, 12, and 52 years old). All elephants in the herd are routinely monitored for EEHV viremia and as such, blood is regularly obtained from them for the purposes of EEHV screening and further study (IACUC approval AN-5182). Two of the elephants (a 5-month-old female and a 3-year-old male) were not studied because obtaining sufficient blood from them to conduct experiments was not feasible. Several of the juvenile elephants are related. In addition, all elephants in this study have evidence of prior EEHV1 infection from shedding, viremia, or serology (Table 2). Basic characteristics of the elephant herd used in these studies can be found in Table 2 and Fig. 8.

### PBMC preparation.

A portion (30 to 40 ml) of venous blood was collected into lithium heparin tubes (BD Vacutainer). Within 2 h of sample collection, the blood was diluted 1:1 at room temperature in RPMI 1640 medium (Thermo Fisher) and centrifuged over a Ficoll-Hypaque gradient (Lympholyte; Cedarlane, Burlington, NC) to isolate PBMCs. The PBMCs were cryopreserved in heat-inactivated fetal bovine serum and 7.5% DMSO and stored in liquid nitrogen until experiments were carried out. When ready to study, the PBMC vials were thawed rapidly in a 37°C water bath and added to prewarmed R-10 complete medium (RPMI + 10% heat-inactivated fetal calf serum [HI-FCS; HyClone] + 1% antibiotic-antimycotic + 1% GlutaMAX) and washed twice prior to counting and use in ELISpot or overnight stimulations.

### Peptide libraries.

Rabies nucleocapsid peptide library was synthesized by GenScript (Piscataway, NJ). MCP and MIE peptide libraries were synthesized by Genemed Synthesis (San Antonio, TX). All other peptide libraries were synthesized by Mimotopes (Melbourne, Australia). With the exception of the rabies nucleocapsid peptide library, all peptides were reconstituted in DMSO at a concentration of 10 mg/ml. Crude rabies NC peptides were reconstituted to a concentration of 200 to 300 μg/ml.

### ELISpot assay.

Ninety-six-well polyvinylidene difluoride membrane ELISpot plates (Merck Millipore, Billerica, MA) were coated overnight with anti-Asian elephant IFN-γ capture antibody (Podiceps, Netherlands) diluted in phosphate-buffered saline (PBS) at 2.5 μg/ml at 4°C. Prior to the addition of cells, plates were washed three times in sterile PBS and blocked in R-10 complete medium for several hours at 37°C. Cells were resuspended in R-10, added to plates at a density of 2 × 10^5^cells/well, and stimulated in a final volume of 200 μl per well at 37°C for 24 h (rabies nucleocapsid pepmix) or 96 h (all other pepmixes). Pepmixes were diluted in R-10 so that each peptide had a final concentration of 1 μg/ml, except for rabies NC, which had a final concentration of 0.2 to 0.4 μg/ml. SEB (2.5 μg/ml; List Biologicals, Campbell, CA) was used as a positive control, while DMSO (Sigma-Aldrich, St. Louis, MO) and survivin (1 ng/ml; JPT, Adlershof, Germany) were used as negative controls. After stimulation, the cells were removed from the plates, and the wells were washed three times in PBS prior to three washes in PBS containing 0.05% Tween. Biotinylated anti-Asian elephant IFN-γ detection antibody (Podiceps) diluted in PBS containing 0.05% Tween and 1% bovine serum albumin (BSA) at 0.625 μg/ml was added to plates for 2 h at room temperature. The plates were then washed four times in PBS containing 0.05% Tween prior to the addition of streptavidin-conjugated alkaline phosphatase diluted 1:1,000 in PBS containing 0.05% Tween and 1% BSA for 1 h at room temperature. Wells were then washed three times in PBS containing 0.05% Tween and three times in PBS prior to the addition of one-step NBT/BCIP substrate solution (Thermo Fisher). The plates were observed for 5 min at room temperature until color development, before the reaction was stopped by adding water to the wells. The wells were washed once more in water and then dried overnight.

### ELISpot counting.

Spot-forming cells (SFCs) were counted on an Immunospot ELISpot reader (CTL, Columbus, OH) using Immunospot software version 5.1.36. The settings were identical for all plates. Counts were then expressed as IFN-γ-positive cells per 10^6^ PBMCs.

### Asian elephant anti-CD8 and anti-CD4 monoclonal antibodies.

CD8 sequence from Loxodonta africana was cloned with an hemagglutinin (HA) tag, into a puc57 vector by using GenScript and expressed in 293T cells (ATCC CRL-3216). For CD4, the predicted transcript was obtained via a tblastn search of an Elephas maximus transcriptome assembly against a set of known mammalian CD4 amino acid sequences. Using BglII and EcoRI restriction enzymes, sequences were subcloned into mscvPURO expression vectors and transfected into 293T cells, in addition to lentivirus VSVG and HIT-60, for the production of infectious transgenic lentivirus. After 48 h, virus was harvested and used to infect mouse L cells. Transgenic lentivirus was combined with L cells in the presence of Polybrene for enhanced infection efficiency. L cells were then grown in Dulbecco modified Eagle medium (+ 10% HI-FCS + 1% antimycotic-antibiotic) containing 2 to 10 μg/ml puromycin, where the concentration of puromycin was increased to 10 μg/ml over a period of several weeks. The cells were plated at 200 cells/well in order to identify puromycin-resistant clones. After 3 to 4 weeks, puromycin-resistant clones were isolated and plated into individual wells of a 24-well plate. Characterization of each clone by Western blotting and flow cytometry enabled identification of those clones that expressed CD8 and CD4 protein, using the HA tag as a target.

The cell lines expressing the highest level of CD8 and CD4 were used to immunize mice, who were subsequently assessed for anti-CD8 and anti-CD4 antibody titers in serum. Mice with the greatest titers were sacrificed, and their spleen cells were fused with myeloma cells to generate potential hybridomas. Hybridoma supernatants were then screened for their ability to bind CD8- and CD4-expressing cell lines versus control cells by using CELLISA.

### Intracellular cytokine staining.

PBMCs (5 × 10^5^) were stimulated with peptides, DMSO, and positive-control SEB overnight at 37°C (5% humidity), where brefeldin A (Thermo Fisher, Waltham, MA) was added after the first 2 h in culture. After stimulation, viability was determined using a Ghost dye Red 780 (Tonbo Biosciences, San Diego, CA). The cells were surface stained with anti-Asian elephant CD8 and CD4 (custom made at the Monoclonal Antibodies Core Facility at the MD Anderson Cancer Center); the cells were phycoerythrin and Pacific blue labeled, respectively, and then lysed and permeabilized using a Cytofix/Cytoperm kit (BD). The cells were then stained for CD3 (Abcam; clone CD3-12) and IFN-γ (Podiceps; clone AE10F4G11, labeled in-house with fluorescein isothiocyanate). An allophycocyanin-labeled anti-rat secondary was added after incubation with primary antibodies in order to detect bound anti-CD3 primary antibody. The cells were then washed and fixed in stabilizing fixative (BD Biosciences). Cells were acquired on a BD FACSCanto II flow cytometer. At least 100,000 events were collected for each sample, determined by BD FACSDiva software. After acquisition, analysis was performed using FlowJo v10 software (TreeStar).

### Statistics.

The statistical comparisons for the continuous data between the experimental groups and the negative control DMSO were performed using two-sample *t* tests with unequal variance assumptions. ELISpot SFC count data were log transformed for stabilizing the variance and approximating the normality assumptions. A *P* value of <0.05 was considered statistically significant. No multiple-comparison procedure was considered. All analyses were performed using GraphPad Prism software.
